# Orthotopic Kidney Transplant as a Fifth Intra-Abdominal Organ after Two Previous Kidney and Two Previous Pancreas Transplants

**DOI:** 10.1155/2022/3823066

**Published:** 2022-06-30

**Authors:** Yazan Al-Adwan, Navdeep Singh, Amer Rajab, Musab Alebrahim, Jayanthan Subramanian, W. Kenneth Washburn, Farjad Siddiqui, Ashley Limkemann, Pranit N. Chotai, Austin Schenk

**Affiliations:** The Ohio State University, Ohio, USA

## Abstract

**Background:**

Patients with more than two prior kidney transplant procedures pose unique surgical challenges. Once both the right and left retroperitoneal spaces have been dissected, intra-abdominal implantation is usually necessary. If the external iliac arteries have been used previously, it is sometimes necessary to use the aorta and vena cava for implantation. Gaining safe exposure in these cases can be complicated by history of prior laparotomy, adhesive disease, and other surgical histories. *Case Presentation*. A 58-year-old female with type 1 diabetes and end-stage renal disease presented for surgical evaluation for kidney transplant. Surgical history was notable for prior simultaneous kidney-pancreas transplant followed by both a living donor kidney transplant and a pancreas after kidney transplant. She had undergone both an allograft nephrectomy and an allograft pancreatectomy and currently had a nonfunctioning kidney in the left retroperitoneal position and a nonfunctioning pancreatic allograft on the right common iliac artery. The entire distal aortoiliac system was surgically inaccessible. She was listed for transplantation, and a cadaveric graft was allocated. Intraoperatively, severe lower abdominal and pelvic adhesions prevented any use of the iliac system. A left native nephrectomy was performed, and the allograft was implanted in the left orthotopic position. The native left renal vein was used for outflow, the donor renal artery was joined end-to-side to the infrarenal aorta, and a uretero-ureterostomy was created. The operation was uneventful. The allograft functioned without delay, and almost one year later, the GFR is approximately 50 mg/dL.

**Conclusion:**

The left orthotopic position can be a good choice for kidney transplant candidates with histories of prior complex lower abdominal surgery.

## 1. Introduction

Renal transplantation is the treatment of choice for patients with end-stage renal disease due to superior short- and long-term survival benefits compared with chronic dialysis treatment. A significant number of wait-listed kidney transplant candidates have medical and surgical comorbidities that increase the complexity of the planned organ transplant. The majority of these patients are older patients with history of hypertension and diabetes mellitus, and frequently, these patients have severe aortoiliac vascular disease. Many of these patients have had multiple prior abdominal organ transplants which increase both immunologic and surgical risk. Scarring and adhesive disease, from prior surgical procedures, increase the risk of perioperative morbidity and mortality. Creative surgical approaches, including consideration of orthotopic kidney transplantation (OKT), are needed in these complex cases.

## 2. Case Presentation

A 58-year-old female with type 1 diabetes diagnosed at the age of eight and complicated by severe retinopathy, neuropathy, and gastroparesis presented for evaluation for her third kidney transplant. She received a simultaneous kidney-pancreas transplant at the age of twenty-four. The pancreas was placed on the right common iliac artery, and the kidney was placed on the right external iliac artery. The pancreas functioned for twenty years, but the kidney was lost due to acute cellular rejection within the first year, and an allograft nephrectomy was performed. At the age of thirty-six, she received a living donor kidney transplant that was placed in the left retroperitoneal space. This functioned for five years and was lost to interstitial fibrosis and tubular atrophy. Lastly, at the age of fifty-one, she received a pancreas-after-kidney during which the prior pancreatic allograft was removed and the new graft was again placed on the right common iliac artery. By the time of the present evaluation, both the second kidney and pancreas transplants had failed and the patient had returned to peritoneal dialysis. This patient had been turned down for repeat transplantation at several other centers due to her complex surgical history. A very thorough physical, serologic, chemical, and radiographic investigation was completed. CPRA was 100%, and blood type was A. After thoughtful consideration by our multidisciplinary transplant candidate selection committee, this patient was listed for transplantation at our center.

**Figure 1:**
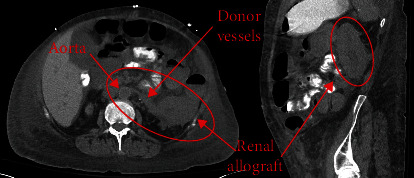
Orthotopic left renal allograft positioning.

After 280 days on the waiting list, a right kidney with KDPI of 22% and a terminal creatinine of 1.3 mg/dL was allocated. The donor had no history of diabetes or hypertension. A physical crossmatch was negative. After thorough explanation of the risks and benefits, the patient was taken to the OR. We began with a midline laparotomy. We encountered extremely dense lower abdominal and pelvic adhesions which effectively prohibited safe access to the iliac system on either side. Our original intention was to transplant onto the left common iliac system; however, it was quickly apparent that this was not a safe approach. Access to the bladder was also hostile. Therefore, we opted for the left orthotopic positioning of this fifth allograft (Figure 1). We completely mobilized the left colon and identified the left renal vein, artery, and native ureter. We clamped and divided the artery, clamped and divided the vein as close to the renal hilum as possible, and divided the native left ureter near the renal pelvis. We passed a probe through the ureter and found that it was patent distally. Methylene blue, which we had previously used to fill the bladder, refluxed through the probed ureter. We then completed the native left nephrectomy.

Prior to implantation of the allograft, we noted a significant size discrepancy between our donor renal vein and the orifice of the native left renal vein. To compensate, we adjusted our clamps such that a semicircle of the gonadal vein and left renal vein was occluded. This required ligation of the adrenal vein and several lumbar branches. This allowed us to open a size-matched venous aperture in the recipient that was formed from both the transected left renal vein and slit placed longitudinally along the gonadal vein. After completing the venous anastomosis, we focused on arterial inflow. We originally intended to sew end-to-end to the cuff of the native left renal artery; however, because of atherosclerosis in this vessel and the anatomy of the right donor kidney, we felt it better to sew directly to the aorta. We dissected out a sufficient length of the infrarenal aorta for clamping. We used a side-biting clamp to partially occlude the aorta, and the anastomosis was completed using the donor Carrel patch. Clamps were removed, and the kidney was reperfused. The color and turgor were excellent. Warm ischemia time was forty-two minutes, and cold ischemia time was 22 hours and 55 minutes. We completed the operation with an uretero-ureterostomy to the left native ureter which we stented with a 6 French 30 cm double J stent.

**Figure 2:**
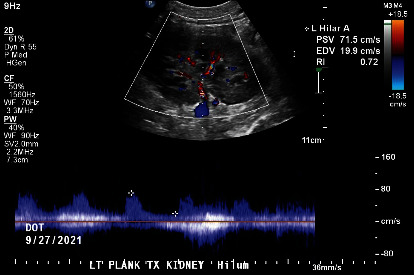
Normal echogenicity and resistive indices of the transplanted kidney as shown by Doppler US.

Postoperatively, Doppler US showed normal echogenicity of the transplanted kidney without hydronephrosis, patent vasculature, and normal resistive indices (Figure 2). The allograft functioned immediately and recipient serum Cr normalized over the first 5 postoperative days without the need for dialysis. The hospital stay was otherwise uneventful, and the patient was discharged home on standard immunosuppression. Almost one year later, the recipient enjoys excellent allograft function with GFR of approximately 50 mg/dL.

## 3. Discussion

Kidney transplant is the treatment of choice for ESRD [1]. Despite advances made with regard to donor and recipient selection, improved immunologic matching, and advances in immunosuppressive therapy, long-term graft survival remains underwhelming (37% graft survival at 15 years). Many patients who experience allograft loss seek retransplantation in hopes of avoiding the high morbidity and mortality associated with return to dialysis [2, 3].

Retransplantation accounts for 13% to 15% of transplants performed annually in the United States and only approximately 5% of those performed in Europe [4]. There are very few reported cases involving more than three intra-abdominal organ transplants in the literature, likely due to the high risk of these medically and surgically complex procedures. Nonetheless, with constant improvements in medical care and the introduction of less toxic immunosuppressive agents, an increasing number of patients are presenting as viable candidates for retransplantation [5].

Traditionally, heterotopic placement is the preferred surgical approach for renal transplantation because this technique reduces complexity, shortens operative time, minimizes ileus, and avoids intra-abdominal adhesions in patients who have had prior abdominal surgery [6, 7]. Patients who have history of multiple previous intra-abdominal organ transplants, extensive pelvic adhesions, caval thrombosis, or severe pelvic atherosclerosis may not be good candidates for heterotopic renal transplantation. In these cases, creative surgical approaches, including orthotopic renal transplantation, must be considered [8].

Gil-Vernet et al. first published the technique of OKT in 1978 and originally described a lumbar approach to left native nephrectomy and subsequent use of the splenic vessels for OKT in patients afflicted with renovascular hypertension [9]. Researchers from the University of Barcelona went on to publish 139 and 233 patient case series of OKT, representing the largest experience in the world [10]. Musquera et al. have observed no significant diferences in graft and patient survival rates between orthotopic and hetertopic kidney transplants in a large series [11]. These series demonstrate both safety and efficacy for this alternative approach to renal transplantation.

## 4. Conclusion

Retransplantation of patients with more than two prior kidney transplants poses unique surgical challenges. Orthotopic kidney transplantation (OKT) is a proven but rarely utilized technique for renal transplantation in patients with a surgically complex lower abdomen or pelvis. In our reported case, OKT was ideal because four previous transplant operations created a hostile and surgically inaccessible lower abdomen. OKT offered means for safe and effective retransplantation in this high-risk candidate.

## Data Availability

There are no data to be declared.

## Abbreviations

ESRD: End-stage renal disease
KDPI: Kidney donor prognostic index
DM: Diabetes mellitus
HTN: Hypertension
CPRA: Calculated panel reactive antibody.
